# Multi-Omic Analysis to Characterize Metabolic Adaptation of the *E. coli* Lipidome in Response to Environmental Stress

**DOI:** 10.3390/metabo12020171

**Published:** 2022-02-11

**Authors:** Thomas Kralj, Madison Nuske, Vinzenz Hofferek, Marc-Antoine Sani, Tzong-Hsien Lee, Frances Separovic, Marie-Isabel Aguilar, Gavin E. Reid

**Affiliations:** 1 School of Chemistry, Bio21 Molecular Science and Biotechnology Institute, The University of Melbourne, Melbourne, VIC 3010, Australia; tom.kralj@unimelb.edu.au (T.K.); madie.nuske@unimelb.edu.au (M.N.); vinzenz.hofferek@unimelb.edu.au (V.H.); msani@unimelb.edu.au (M.-A.S.); fs@unimelb.edu.au (F.S.); 2 Department of Biochemistry and Molecular Biology, Monash University, Clayton, VIC 3800, Australia; john.lee@monash.edu (T.-H.L.); mibel.aguilar@monash.edu (M.-I.A.); 3 Department of Biochemistry and Pharmacology, The University of Melbourne, Melbourne, VIC 3010, Australia

**Keywords:** *E. coli*, lipidome, proteome, environmental stress, mass spectrometry, ion mobility, photodissociation

## Abstract

As an adaptive survival response to exogenous stress, bacteria undergo dynamic remodelling of their lipid metabolism pathways to alter the composition of their cellular membranes. Here, using *Escherichia coli* as a well characterised model system, we report the development and application of a ‘multi-omics’ strategy for comprehensive quantitative analysis of the temporal changes in the lipidome and proteome profiles that occur under exponential growth phase versus stationary growth phase conditions i.e., nutrient depletion stress. Lipidome analysis performed using ‘shotgun’ direct infusion-based ultra-high resolution accurate mass spectrometry revealed a quantitative decrease in total lipid content under stationary growth phase conditions, along with a significant increase in the mol% composition of total cardiolipin, and an increase in ‘odd-numbered’ acyl-chain length containing glycerophospholipids. The inclusion of field asymmetry ion mobility spectrometry was shown to enable the enrichment and improved depth of coverage of low-abundance cardiolipins, while ultraviolet photodissociation-tandem mass spectrometry facilitated more complete lipid structural characterisation compared with conventional collision-induced dissociation, including unambiguous assignment of the odd-numbered acyl-chains as containing cyclopropyl modifications. Proteome analysis using data-dependent acquisition nano-liquid chromatography mass spectrometry and tandem mass spectrometry analysis identified 83% of the predicted *E. coli* lipid metabolism enzymes, which enabled the temporal dependence associated with the expression of key enzymes responsible for the observed adaptive lipid metabolism to be determined, including those involved in phospholipid metabolism (e.g., ClsB and Cfa), fatty acid synthesis (e.g., FabH) and degradation (e.g., FadA/B,D,E,I,J and M), and proteins involved in the oxidative stress response resulting from the generation of reactive oxygen species during β-oxidation or lipid degradation.

## 1. Introduction

The dynamic remodelling of membrane phospholipid compositions occurs in response to a variety of exogenous environmental stresses, including exposure to antibiotics, antimicrobial peptides, changes in temperature, osmolarity, pH, alcohol, reactive oxygen species, and nutrient depletion [1,2,3,4,5,6,7,8,9]. This adaptive response can facilitate the elimination of and/or development of resistance to the stress stimuli, thereby enhancing bacterial resilience and survival. Modifications to bacterial membrane phospholipid compositions that occur as part of exogenous stress responses include alterations in the relative concentrations of cationic phosphatidylethanolamine (PE) and anionic phosphatidylglycerol (PG) or cardiolipin (CL) lipids, modification of anionic PG head groups to form cationic lysyl-phosphatidylglycerol (lysyl-PG), remodelling of fatty acyl-chain lengths, alterations of the degree of unsaturation and the incorporation of odd-numbered chain length fatty-acyls containing iso- or anteiso-branched chain or cyclopropyl modifications [9,10,11,12,13,14]. The effect of these modifications may be categorised in terms of their impact on the net surface charge, thickness, fluidity and ordering of the membrane, as well as membrane curvature and domain formation [15,16,17]. Understanding the global lipid membrane’s compositional changes that occur upon exposure to environmental stress, or the individual lipid changes that may occur upon exposure to a particular stress, can therefore provide insights into the underlying mechanisms responsible for the adaptive response(s).

To date, various ‘omics’-based studies investigating bacterial adaptations have contributed to a greater understanding of the changes in the proteome, metabolome and lipidome of bacterial cells in response to exogenous stresses, or such changes that lead to resilience against such stresses including the development of antibiotic resistance [16,18,19,20,21,22,23,24,25,26]. For example, mass spectrometry (MS)-based lipidomics has previously been utilised to identify changes in lipid composition and structure that are associated with exogenous stress adaptations in a number of bacterial species [16,21,22,23,24,25,26]. In a lipopeptide antibiotic daptomycin-resistant strain of Gram-positive *Staphylococcus aureus*, where whole-genome sequencing identified a loss-of-function mutation of PG synthase as well as mutations in genes affecting fatty acid biosynthesis and cell wall metabolism, decreased PG, CL and lysyl-PG levels were all observed, along with a shift towards longer fatty-acyl chains and decreases in the abundance of lipids containing odd-numbered fatty-acyl chains [21]. Acyl-chain remodelling, including increased unsaturation, decreased levels of cyclopropane lipids and changes in the ratios of iso- to anteiso-branched acyl-chains species in response to the antimicrobial agent naringenin and the antimicrobial peptide apidaecin 1b, have also been reported for Gram-negative *Escherichia coli* (*E. coli*) [16,22].

During the exponential growth phase (EGP), *E. coli* utilises fermentable sugars from the surrounding growth medium as its primary carbon source. Under nutrient depletion stress conditions, however, it transitions into the stationary growth phase (SGP), where lipids are utilised as an alternative carbon source to maintain cell growth and survival. Under these conditions, the bacteria can also experience other environmental stresses, including changes in osmolarity, decreased pH and an increase in the concentration of reactive oxygen species (ROS) [7,12,26,27,28,29]. These stresses result in *E. coli* undergoing significant changes in the composition and structure of its membrane lipids, including increased CL, and increases in the cyclopropyl modification content of phospholipid fatty acyl chains [9,12,30]. Under SGP conditions, lipid adaptation in *E. coli* also results in increased resilience to antibiotics compared with that seen in EGP, suggestive of bacterial ‘cross-protection’ resulting from common alterations occurring within certain lipid metabolism pathways as a response to different stresses [31,32,33,34].

Unfortunately, lipidome analysis strategies involving characterisation of the changes in bacterial lipid composition and structure are not necessarily sufficient to fully describe the adaptations that occur in lipid metabolism in response to a particular environmental stress, as changes in the lipidome may result from alterations in the expression or activity of a specific enzyme, or a collection of enzymes, involved in a particular metabolic pathway. Thus, ‘multi-omics’ analysis approaches (e.g., lipidomics and proteomics), which enable the identification, characterisation and quantification of both lipids and proteins extracted from a single sample [35,36,37,38,39], may provide a more holistic understanding of bacterial lipid membrane adaptation mechanisms, including elucidation of the relationship between a specific stress and alterations in individual lipids and specific lipid metabolism enzymes [40,41].

Here, with *E. coli* as a well characterised model system, an integrated sample extraction, sample preparation and MS-based workflow for ‘multi-omics’ lipidome and proteome analysis (Supplementary Figure S1) is described, which enables comprehensive characterisation of the lipid and protein compositions in *E. coli* under nutrient depletion stress conditions, i.e., between EGP and SGP conditions, and which enables the interrelationship and temporal dependence between changes in lipid classes or specific lipids and the specific lipid metabolism enzymes responsible for their formation to be determined.

## 2. Results and Discussion

### 2.1. Characterisation of the E. coli Lipidome under Exponential Growth Phase (EGP) and Stationary Growth Phase (SGP) Conditions

**Figure 1:**
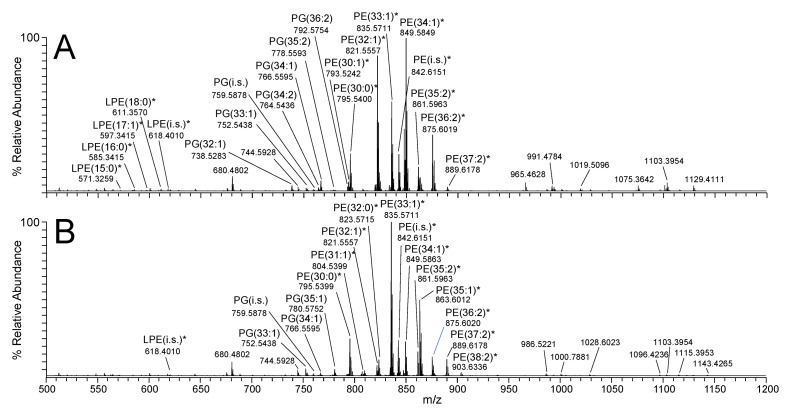
Positive ionisation mode (+ve) ‘shotgun’ nESI-UHRAMS spectra of crude derivatised lipid extracts from *E. coli* grown under (**A**) EGP (O.D. = 1) and (**B**) SGP (O.D. = 4.6) conditions. * indicates a derivatised PE lipid.

**Figure 2:**
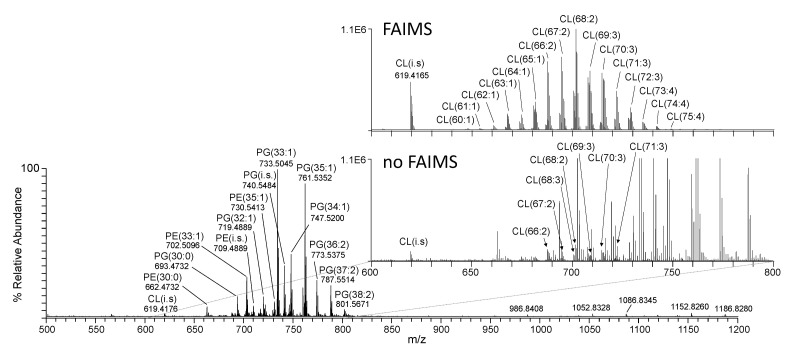
Negative ionisation mode (-ve) ‘shotgun’ nESI-UHRAMS spectra of a crude non-derivatised lipid extract from *E. coli* grown under SGP conditions. The lower inset shows an expanded region (600–800 *m*/*z*) of the mass spectrum. The upper inset shows an expanded region (600–800 *m*/*z*) of the mass spectrum acquired using nESI-FAIMS-UHRAMS on the same sample.

Figure 1A,B shows representative mass spectra acquired from the positive ionisation mode (+ve) ‘shotgun’ nano-electrospray (nESI)-MS spectra of crude derivatised lipid extracts from *E. coli* grown under EGP and SGP conditions, respectively. Derivatisation using S,S’-dimethylthiobutanoylhydroxysuccinimide ester (DMBNHS) and iodine/methanol (MeOH) enabled the resolution of potential isomeric mass overlaps between PE and PG lipids from any phosphatidylcholine and phosphatidylserine lipids that could potentially be present, respectively, while also increasing the ionisation and detection sensitivity of aminophospholipids, as well as enabling the resolution of potential plasmalogens fromplasmanyl lipids [42,43,44,45]. This approach, therefore, provides a simple strategy for the unambiguous assignment of these lipid species at the sum-composition level (19 PE and 14 PG lipids) based only on their accurate mass values determined using ultrahigh resolution accurate mass spectrometry (UHRAMS), without the need for chromatographic separation prior to MS analysis. Negative ionisation mode was used for the analysis of cardiolipins, via observation of their doubly deprotonated [M−2H]^2−^ precursor ions. Figure 2 shows an example of the spectra acquired from *E. coli* grown under SGP conditions. The lower inset in this figure shows a region of the mass spectrum from 600 to 800 *m*/*z* from which several ions could be tentatively assigned as cardiolipins based on the characteristic 0.5 *m*/*z* spacing between their isotopes. However, due to the low relative abundance of these ions (approx. 2% of the base peak in the spectrum) and the congestion caused by the presence of many isobaric mass PE and PG lipids at similar *m*/*z* values, definitive annotation of these lipids was quite limited. In contrast, the upper inset in Figure 2 shows a region of the mass spectrum from 600 to 800 *m*/*z* acquired using nESI-high-field asymmetric-waveform ion-mobility spectrometry (FAIMS)-MS on the same sample, where all singly charged ions were filtered out, allowing the CL species to be clearly observed [46,47]. In addition, the signal intensity for these CL lipids was significantly increased by approximately fivefold due to their selective transmission and accumulation. Supplementary Figure S2 contains a comparable nESI-FAIMS-MS spectrum from the EGP sample. As a result of incorporating FAIMS into the analysis workflow, 34 CL species could be unambiguously identified at the sum-composition level within the EGP and SGP samples, whereas only 22 could be tentatively assigned in its absence.

**Figure 3:**
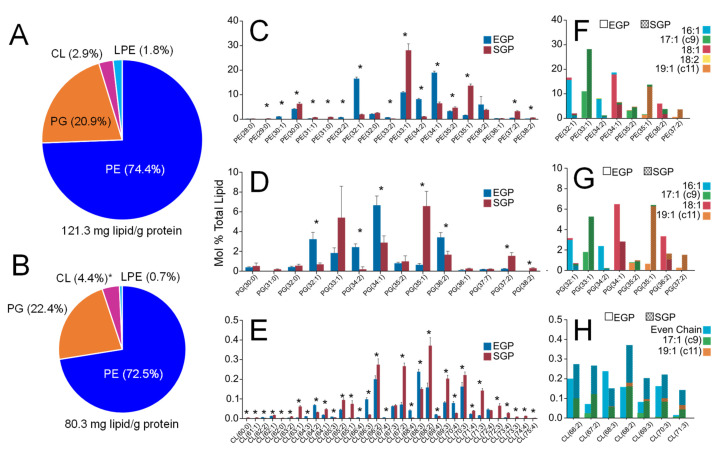
Summary of the lipidome composition of *E. coli* observed under EGP and SGP conditions. Mol% total lipid for (**A**) EGP and (**B**) SGP at the lipid class level of annotation; for (**C**) PE, (**D**) PG and (**E**) CL lipid species at the ‘sum composition’ level of annotation; and for (**F**) PE, (**G**) PG and (**H**) CL lipid species at the ‘molecular lipid’ level of annotation. The data in Panel E were obtained using FAIMS, while the data in panels (**F**–**G**) were obtained using CID- or 213 nm UVPD-MS/MS. * Denotes lipids which were present at significantly different abundances between EGP and SGP conditions (n = 5) as determined by a Student’s *t*-test with Holm–Šidák’s post hoc method (*p*-value ≤ 0.01).

Following data analysis and quantitation against the respective internal lipid standards, a total lipid content of 121.3 mg and 80.3 mg of PE, PG and CL lipid per g of protein was determined for the EGP and SGP cells, respectively (Figure 3A,B, respectively), with the most abundant lipid class being PE (74.4% and 72.5% in EGP and SGP, respectively), followed by PG (20.9% and 22.4%). The observed decrease in total lipid content under SGP conditions is likely to be due to some of the cellular population entering into the cell death phase, as well as a loss of total lipid content due to catabolic lipid metabolism in response to nutrient depletion stress. Between the EGP and SGP conditions, the abundance of the PE and PG lipid classes did not undergo any major changes, while CL underwent a significant increase from 2.9% to 4.4%. However, there was a significant change observed in the abundances of individual PE, PG, and CL lipids within each class, as determined at the sum-composition level of analysis (Figure 3C–E). Lipids in the SGP cells underwent a consistent net shift towards species containing odd-numbered acyl-chain lengths, with a corresponding decrease in those containing even-numbered acyl chains, for PE, PG and CL species. For example, while PE(32:1) and PE(34:1) were the two most abundant lipid species in the EGP lipidome (Figure 3C), PE(33:1) and PE(35:1) were the most abundant in the SGP lipidome. A similar pattern was observed for PG lipids, e.g., a shift from PG(32:1) and PG(34:1) in the EGP to PG(33:1) and PG(35:1) in the SGP (Figure 3D). The shift from even- to odd-numbered chain lengths is more evident when the ratio of odd- to even-numbered chain lengths was assessed for the EGP and SGP samples. Under EGP conditions, the abundance of even-numbered chain lengths for all lipids was 2.3-fold greater than that of odd-numbered chain length lipids, with PE, PG, and CL species with even-numbered chain lengths being present at a 3.3-, 4.5-, and 3.3-fold greater abundance, respectively. However, the abundance of odd-numbered chain lengths for all lipids in the SGP was 2.7-fold greater than that of even-numbered chain length lipids, with PE and PG lipids with odd-numbered chain lengths being present at a 2.3- and 2.1-fold greater abundance, respectively.

Negative ionisation mode collision induced dissociation—tandem mass spectrometry (CID-MS/MS) of the most abundant PE, PG and CL lipids confirmed a shift from phospholipids containing (16:1) and (18:1) as the most abundant acyl chains in the EGP lipidome, consistent with prior reports in the literature, to those containing (17:1) and (19:1) acyl chains in the SGP lipidome (Figure 2F–H). An additional example is shown in Supplementary Figures S3 and S4, for the [M−H]^−^ precursor ions of PE(38:2) (770.56 *m*/*z*) from *E. coli* grown under EGP and SGP conditions, respectively. Although it was one of the least abundant PE lipids observed in both samples, this apparently ‘even’-numbered lipid underwent the greatest fold change increase between EGP and SGP conditions, suggesting that it could contain two fatty acyl groups with odd-numbered chain lengths. Under EGP conditions, this lipid was found to comprise four individual species, predominantly consisting of PE(18:1_20:1), along with PE(16:0_22:2), PE(16:0_22:2) and PE(19:1/19:1), all at a much lower relative abundance. However, under SGP conditions, PE(19:1/19:1) became the predominant lipid species. A similar trend was seen for CL species, where the apparently ‘even’-numbered CL(68:2), CL(68:2) and CL(66:2) species in the SGP conditions were found to contain two acyl chains with odd-numbered chain lengths of (17:1) or (19:1), which were all absent in the EGP samples. Through this CID-MS/MS analysis, the number of lipid species identified for PE and PG was increased to 29 and 20, respectively, while FAIMS coupled with CID-MS/MS enabled the number of identified CL species to be increased to 46.

**Figure 4:**
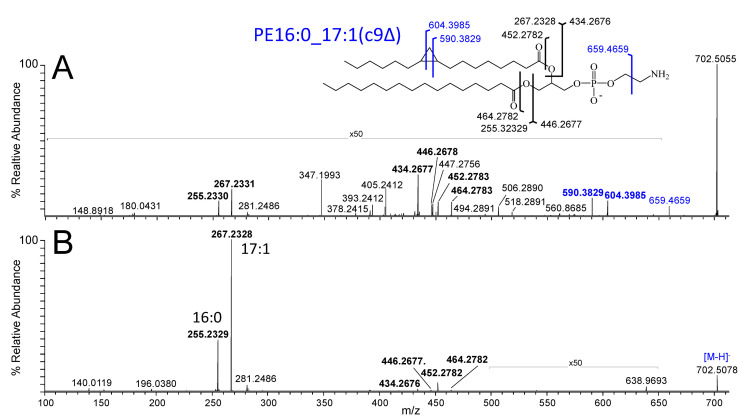
Negative ionisation mode spectra obtained by (**A**) 213 nm UVPD-MS/MS and (**B**) CID-MS/MS for structural characterisation of the [M−H]^−^ precursor ion of *E. coli* PE(33:1) observed at 733.5045 *m*/*z* in Figure 2 as predominantly containing PE16:0_17:1(c9Δ) under SGP conditions. The structure shown in the inset indicates the assigned bond cleavage sites for the major product ions. Product ions labelled in blue text are unique to UVPD.

Previous reports have demonstrated that as *E. coli* enters into SGP conditions upon encountering nutrient depletion stress conditions, its lipid membrane is remodelled via the addition of cyclopropyl groups to monounsaturated fatty acyl-containing phospholipids [48,49,50], resulting in a shift from even-numbered acyl chains to odd-numbered acyl chain species containing one double bond equivalent. Although the presence of a cyclopropyl group versus other potential odd-numbered acyl chain species (e.g., monounsaturated linear or branched chain species) cannot be differentiated using conventional CID-MS/MS methods, the identification of lipids containing cyclopropyl groups can be readily determined using negative ionisation mode 213 nm ultraviolet photodissociation (UVPD)-MS/MS via the observation of unique diagnostic product ions, as previously described by Blevins et al. [51] and Macias et al. [52]. Examples for PE(33:1) (733.5045 *m*/*z* in Figure 2) and PG(37:2) (787.5514 *m*/*z* in Figure 2) are shown in Figure 4A (the corresponding CID-MS/MS spectrum is shown in Figure 4B) and Supplementary Figure S5, respectively, under SGP conditions. The structures shown in the insets in these figures indicate the assigned bond cleavage sites for the major product ions. In Figure 4A, the pair of product ions at 604.3985 *m*/*z* and 590.3829 *m*/*z*, differing in mass by 14 Da, enables the presence of the cyclopropyl group as well as its location at the c9Δ (i.e., the n-7) position along the (c17:1) acyl chain of the PE(33:1) lipid, i.e., PE(16:0_17:1(c9Δ)) to be unambiguously assigned. A similar pair of product ions at 675.4242 *m*/*z* and 689.4398 *m*/*z* unambiguously localised the cyclopropyl group to the c11Δ (i.e., n-9) position of the (c19:1) acyl chain within the PG(37:2) lipid, i.e., PG(18:1_19:1(c11Δ)). Unfortunately, no information was obtained from the negative ionisation mode UVPD-MS/MS experiment to localize the position of the C=C double bond within the (18:1) acyl chain of this and other unsaturated lipids seen in this study. However, the locations of acyl chain C=C double-bonds in *E. coli* lipids have previously been reported by using UVPD-MS/MS analysis in positive ionisation mode, where the sites of unsaturation were conserved (i.e., only one C=C location was preferred) at the c9 position for C16 chains and at the C11 position for C18 chains. Thus, as it is these sites that are converted to cyclopropyl groups via the action of the Cfa enzyme, it was not necessary in this study to verify the double-bond locations or to contrast the changes in C=C double-bond positions between the lipidomic information of EGP and SGP [53,54]. Finally, UVPD-MS/MS of each of the ions indicated in Figure 3 revealed no heterogeneity with respect to the locations of the cyclopropyl groups within the (c17:1(9Δ)) or (19:1(11Δ)) acyl chains.

### 2.2. Proteomic Analysis of E. coli

**Figure 5:**
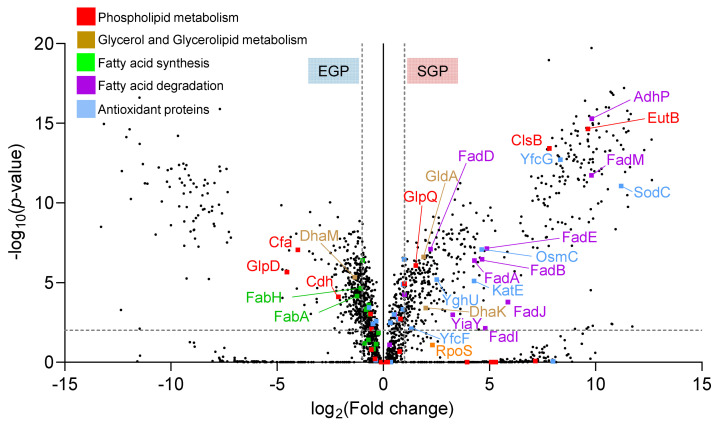
Volcano plot of altered protein abundances from whole-cell extracts of *E. coli* grown under EGP and SGP conditions, using nLC-DDA-MS/MS proteome analysis. Selected proteins associated with phospholipid metabolism (red), fatty acid synthesis (green), fatty acid degradation (purple), and antioxidant proteins (blue) are highlighted. Dashed lines indicate a twofold change in abundance and *p* < 0.01. The full names and abbreviations of the enzymes labelled in this figure are shown in Supplementary Table S1.

**Figure 6:**
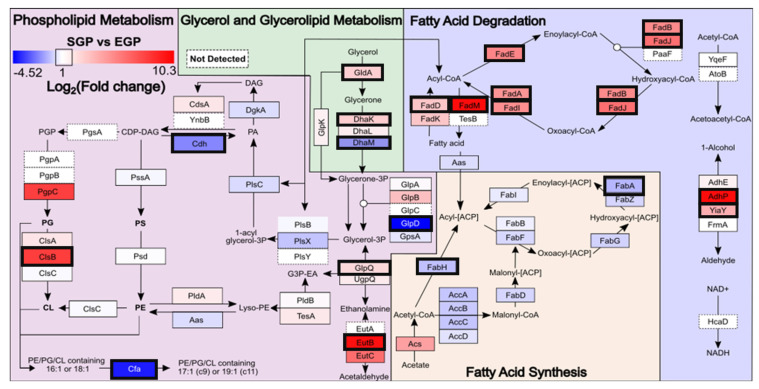
Pathway of *E. coli* lipid metabolism annotated to show alterations in lipid metabolism-associated protein abundances between EGP and SGP conditions. A heatmap is used to show proteins with increased abundance in either the EGP (blue) or SGP (red). Proteins whose abundance changes are statistically significant are annotated with a bold black box. Proteins not observed are annotated with a dashed white box. The full names and abbreviations of the enzymes labelled in this figure are shown in Supplementary Table S1.

The results described above indicate that remodelling of one part of the phospholipid metabolism pathway (a significant increase in total CL), as well as extensive cyclopropyl fatty acid incorporation, occurs in response to *E. coli* encountering nutrient depletion stress conditions and therefore entering the SGP. To obtain insights into the key enzymes responsible for, and the temporal dependence on, this lipid metabolism pathway remodelling, data-dependent acquisition (DDA) LC-MS/MS proteomic analysis with label-free quantitation was performed on the whole-cell protein extracts for *E. coli*. under EGP and SGP conditions. From this analysis, 2,184 proteins were identified in total (81% common to both conditions), covering 51% of the entire expected *E. coli* proteome (Supplementary Figure S6). Of these, 439 proteins were found to be significantly increased and 365 significantly decreased in abundance in the SGP compared with the EGP (Figure 5). Of particular relevance to this study, 64 of the 77 known lipid metabolism-associated proteins were identified, representing 83% coverage of the expected *E. coli* lipid metabolism proteome. Of these, 14 proteins (Adhp, FadM, EutB, cardiolipin synthase B (ClsB), FadJ, FadE, FasI, FadB, FadA, YiaY, FadD, DhaK, GldA and GlpQ) were significantly increased in abundance, while six (cyclopropane-fatty-acyl-phospholipid synthase (Cfa), beta-ketoacyl-acyl carrier protein synthase III (FabH), FabA, DhaM, Cdh and GlpD) were significantly decreased in abundance under SGP conditions compared with the EGP. These proteins, annotated according to their functions in phospholipid metabolism, glycerol and glycerolipid metabolism, fatty acid synthesis and fatty acid degradation are shown in Figure 6.

Within the phospholipid metabolism pathway, one of the most highly upregulated proteins was ClsB, which was only detected in the SGP samples, consistent with previous reports indicating that it is only expressed under SGP conditions [55], as well as with the observations from the lipidome analysis above that total CL was significantly increased in the SGP compared with the EGP. The increased synthesis of CL under SGP conditions has previously been reported and is associated with increased resistance to osmotic stress, enhanced oxidative phosphorylation, ATP synthesis and cell survival [12,29,56]. Increased CL has also previously been proposed as a contributing factor to the increased resilience of SGP cells to antimicrobials compared with those from the EGP [13].

According to the characterisation of PE, PG, and CL from the *E. coli* lipid extracts, it is evident that a large portion of unsaturated fatty acyl chains were converted to cyclopropyl-containing fatty acyl species in the SGP (Figure 3). The presence of cyclopropyl-containing acyl-chains within membrane phospholipids are known to be associated with increased resilience to environmental stresses and antibiotic resistance in bacteria and other microorganisms [14,31,57]. At the proteome level, one of the most highly downregulated proteins within the phospholipid metabolism pathway in SGP versus EGP conditions was Cfa, which is responsible for the conversion of C=C double bonds in unsaturated phospholipid fatty acyl chains to cyclopropyl groups. This is consistent with previous reports where, after the initial expression of Cfa in response to environmental stress (nutrient depletion), it rapidly proteolytically degraded in SGP [50,58]. Therefore, the increased expression of Cfa determined by proteome analysis in the EGP is temporally separate from (i.e., precedes) the observation of an increased abundance of cyclopropyl-containing lipid products in the SGP, as determined by lipidome analysis.

Under EGP conditions, cell replication requires a continual uptake of carbon nutrients to produce energy, and membrane lipids [59]. Therefore, cells transition to SGP conditions when the primary carbon sources such as fermentable sugars are depleted [27]. As a result, cell division ceases and the metabolism is altered to adapt to nutrient depletion stress. To maintain cellular energy homeostasis, lipids can be used as an alternate carbon source; therefore, lipid degradation and β-oxidation provide bacteria with energy in the SGP for survival [60]. Consistent with this, the SGP proteome exhibited an significant overall increase in abundance for the majority of proteins involved in fatty acid degradation compared with the EGP, while all proteins involved in the fatty acid synthesis pathway trended toward a decrease in abundance under SGP conditions, with two proteins, namely FabH and FabA, reaching significance (Figure 3). Similar what was discussed above for Cfa, the upregulation of FabH is a determining factor in branched-chain fatty acid biosynthesis under EGP conditions and is likely to be responsible for the observed highly significant increase in abundance, despite the low absolute abundance, of odd-numbered saturated branched-chain fatty acyl chains containing PE(29:0), PE(31:0) and PG(31:0) lipids under SGP conditions (Figure 3) [61,62].

In the SGP, *E. coli cells* experience high internal stress, causing them to be susceptible to increased oxidative stress from ROS as well as pH, osmolarity and temperature [63,64,65,66]. ROS are generated from various metabolic processes, including the β-oxidation resulting from lipid degradation. Previous studies have shown that the stress response in *E. coli* is mediated by the RNA polymerase sigma factor (RpoS) protein [57], resulting in a cascade leading to an upregulation of various antioxidant-associated proteins as a protective mechanism, which provide resilience against antibiotics and other stresses [32,33,34,67] and are characteristic of *E. coli* in the SGP [65,67]. Here, a 4.9-fold increase was observed for RpoS in the SGP proteome, albeit not statistically significant (Figure 5). However, proteomic analysis resulted in the identification of 18 proteins with an antioxidant function, including some for the decomposition of ROS [6,66], for which six proteins (superoxide dismutase (SodC), osmotically inducible protein C (OsmC), catalase hydroxy-peroxidase II (KatE), the disulphide-bond oxidoreductases (YfcG and YghU), and glutathione S-transferase (YfcF)), showed significantly increased abundance in the SGP compared with the EGP (Figure 5).

## 3. Materials and Methods

### 3.1. Materials

Internal lipid standards were purchased from Avanti Polar Lipids and were combined to produce an *E. coli* internal standard mixture (Supplementary Table S2). Ammonium bicarbonate and HPLC grade chloroform (CHCl_3_) were purchased from Ajax Finechem. Ammonium formate and sodium chloride were purchased from ChemSupply. Butylated hydroxytoluene (BHT), chloroacetamide (CAA), iodine, isopropanol (IPA), MS grade MeOH and acetonitrile (ACN), phosphate-buffered saline (PBS), phosphoric acid, sodium dodecyl sulfate (SDS), triethylamine (TEA), triethylammonium bicarbonate (TEAB) and tris(2-carboxyethyl)phosphine (TCEP) were from Merck, and 0.15 mm zirconium oxide beads were purchased from Next Advance. The yeast extract and tryptone were from Oxoid. MS grade Pierce trypsin protease and isopropanol were purchased from Thermo Fisher, and ^13^C_1_-DMBNHS was synthesised as previously described [43,44].

### 3.2. E. coli Cell Culture

The *E. coli* BL21 strain was cultured overnight in 250 mL of lysogeny broth (LB) (5 g/L NaCl, 5 g/L yeast and 10 g/L tryptone) at 37 °C at 250 rpm in a 500 mL Erlenmeyer flask. After that, 1.5 mL of this overnight culture was used to inoculate 150 mL of LB, which was then cultured under the same growth conditions. Cells were harvested when an optical density (OD) of 1 at 600 nm was reached (i.e., the EGP) and once the cells had reached SGP conditions (OD of 4.6), after which no further increase in OD was observed. OD was checked periodically using 1 mL of culture and an OD600 DiluPhotometer (Implen).

### 3.3. Monophasic Lipid Extraction and Derivatisation of Aminophospholipids and Plasmalogen-Containing Lipids

From the *E. coli* culture, 50 mL of EGP culture was transferred to a 50 mL centrifuge tube (Falcon) and spun at 5000× *g* for 10 min at 4 °C. The supernatant was discarded, and the cells were washed with 40 mL of PBS and pelleted again using the previously mentioned centrifugation conditions. The cell pellet was then washed and pelleted twice more, resuspended in 10 mL PBS and separated into five 1 mL technical replicate aliquots in 1.5 mL microcentrifuge tubes (Eppendorf). The five replicates were pelleted at 8000× *g* for 5 min at 4 °C, after which the supernatant was removed, then snap-frozen in liquid nitrogen and freeze-dried. Eleven millilitres of SGP culture was diluted to 50 mL to ensure an equal number of cells was harvested for both the EGP and SGP samples, then the cells were washed and pelleted as above. The protein content for each sample replicate was measured with a Pierce™ BCA Protein Assay kit (Thermo Fisher) with the EGP replicates having a mean protein content of 478.4 ± 43.2 µg and the SGP replicates having a mean protein content of 487.4 ± 26.7 µg.

Lipids were subjected to monophasic extraction as reported previously [42,43]. To the freeze-dried cells, a 100 µL scoop of 0.15 mm zirconium oxide beads was added with 200 µL of 60% v/v aqueous MeOH containing 0.01% w/v BHT and 50 µL of the internal lipid standard mix in CHCl_3_. The samples were homogenised using a Bullet Blender storm 24 (Next Advance) on Speed 8 for 30 s, which was repeated twice, with the sample being cooled on ice between each homogenisation run. After homogenisation, 120 µL of H_2_O, 250 µL of CHCl_3_ containing 0.01% w/v BHT and 420 µL of MeOH containing 0.01% w/v BHT was added to the homogenate solution to achieve a 0.74:1:2 H_2_O:CHCl_3_:MeOH extraction solution. The samples were then vortexed for 1 min and shaken at 1400 rpm for 30 min in a Thermomixer compact mixer (Eppendorf). The samples were pelleted at 10,000 *g* for 15 min, and the supernatant was transferred to a 2 mL microcentrifuge tube (Eppendorf). The remaining pellet was re-extracted, for which 100 µL of H_2_O and 400 µL of 1:2 CHCl_3_:MeOH containing 0.01% w/v BHT was added to the pellet, which was homogenised again and then pelleted by centrifugation. The supernatant from the re-extraction was then combined with the supernatant from the first extraction. Both the remaining pellets and the combined supernatant lipid extracts were dried using a miVac centrifugal concentrator (Genevac) until completely dry. Once dry, the supernatants were reconstituted in 500 µL of IPA:MeOH:CHCl_3_ (4:2:1) containing 0.01% w/v BHT. For analysis of underivatised samples, 10 µL of each sample was aliquoted onto a 96-well twin.tec PCR plate (Eppendorf), dried and then reconstituted in 40 µL of IPA:MeOH:CHCl_3_ (4:2:1) containing 20 mM ammonium formate.

Functional group selective derivatisation of aminophospholipids and plasmalogen lipids to resolve potential isomeric mass overlaps was performed as previously described [43,44,45]. To derivatise the aminophospholipids, a 10 µL aliquot of each sample was aliquoted into a 96-well round-bottom Multi-Chem plate (Whatman) and dried using centrifugal vacuum evaporation for 15 min. The dried samples were reconstituted in 40 µL of CHCl_3_ containing 60 µM TEA and 67 µM ^13^C_1_-DMBNHS, and the plate was sealed and incubated for 30 min at room temperature on an orbital shaker at 150 rpm. The plate was then unsealed and dried using centrifugal vacuum evaporation. To derivatise the plasmalogens, 40 µL of ice-cold CHCl_3_:MeOH (2:1) containing 177 µM I_2_ and 667 µM ammonium carbonate was added to each sample then incubated on ice for 5 min. The samples were dried again, washed three times with 40 µL of 10 mM aqueous ammonium carbonate before being dried again, then reconstituted for lipidome analysis in 40 µL of IPA:MeOH:CHCl_3_ (4:2:1) containing 20 mM ammonium formate.

### 3.4. nESI-UHRAMS, CID-MS/MS and 213 nm UVPD-MS/MS Lipid Analysis

Lipid extracts were introduced to an Orbitrap Fusion Lumos mass spectrometer (Thermo Fisher, San Jose, CA, USA) via direct infusion using a Triversa Nanomate nESI source (Advion) operating with an ionisation voltage of 1.40 kV and a gas pressure of 0.30 psi. MS analysis was performed with a 1.5 min acquisition time over a *m*/*z* range of 350–1600 with a mass resolving power of 500,000, and with the RF lens at 10% and 2 microscans. Underivatised samples were analysed in both the positive and negative ionisation modes, while derivatised samples were only analysed in the positive ionisation mode. MS/MS analysis using CID was performed on selected phospholipid precursor ions (underivatised samples) to characterise the fatty acyl chain lengths and the degree of saturation, using a collision energy of 28%, an activation time of 10 ms and a mass resolving power of 120,000, with an isolation window of 1 *m*/*z* and a 1 min acquisition time. Next, 213 nm UVPD of selected phospholipid precursor ions (underivatised samples) was used to identify and localize the position of cyclopropyl groups in the phospholipid fatty acyl chains, using an 800 ms activation time, a mass resolving power of 120,000, a mass isolation window of 1 *m*/*z* and a 5 min acquisition time.

### 3.5. Cardiolipin Analysis Using FAIMS-MS

For the identification and characterisation of CL species, sample introduction and analysis were performed in the negative ionisation mode, similar to that described above but with filtering/enrichment achieved using FAIMS (FAIMS Pro, Thermo Fisher, Waltham, MA, USA) operating at a standard resolution with a static gas flow and a compensation voltage of 61 V (optimised for the transmission of cardiolipin [M−2H]^2−^ precursor ion charge states) [45,46]. Spectra were acquired for 2.0 min with a *m*/*z* range of 400–1200.

### 3.6. Lipid Identification and Quantification

Analysis of the lipidomics data was performed using LipidSearch 5.0.58α (Mitsui Knowledge Industry, Tokyo, Japan) with automated peak peaking, correction of ^13^C isotope abundances and identification of lipids at the ‘sum composition’ level of annotation using a user-generated lipid database as previously described [25,47]. The database included all major *E. coli* lipid classes and accounted for deuterated internal lipid standards and mass shifts as a result of derivatisation with ^13^C_1_-DMBNHS and/or iodine/MeOH. The precursor mass tolerance was set at 3.0 ppm with a precursor ion intensity threshold set at 3 times the observed noise intensity, the correlation threshold (%) was 0.3, the isotope threshold (%) was 0.1 and the max isotope number was set at 1. Peak detection was set to the profile and merge mode to average. Semi-quantitative analysis of endogenous PE, PG and CL species was achieved by comparing the peak areas of the identified lipids of interest with the peak area of their respective internal standards. The LipidSearch output was further processed using an in-house developed R script for visualisation and quantification of the identified lipids. Zero filling was performed for lipids which were not detected in a replicate by using a value 1/3 of the minimum value from the entire dataset. Further filtering was performed to remove any lipids which were not present in all replicates in either the EGP or SGP sample groups, those that had a CV > 50%, or those that represented less than 0.1% of the total lipid abundance.

### 3.7. Proteomic Sample Preparation

Protein pellets from the monophasic lipid extraction were resuspended in 5% w/v SDS in PBS, then shaken at 1400 rpm for 30 min at 37 °C prior to centrifugation at 10,000× *g* for 5 min to pellet any remaining insoluble components. Next, 50 μg of protein from each sample was prepared for analysis using S-Trap (ProtiFi) mini-columns, with sample preparation performed as per the manufacturer’s protocol. Reduction and alkylation were performed in 2 mL microcentrifuge tubes (Eppendorf) with 10 mM TCEP and 40 mM CAA (5 min incubation at 99 °C). After reduction and alkylation, the SDS solution was acidified with 12% v/v phosphoric acid to a final concentration of 1.2% v/v phosphoric acid. The acidified solution was then diluted sevenfold with an S-trap binding buffer (100 mM TEAB in 90% v/v aqueous MeOH) to create a colloidal protein suspension. The samples were then loaded onto the S-trap mini-columns and centrifuged at 4000× *g* for 30 s, trapping the proteins in the S-trap column matrix. The trapped proteins were washed 4 times with 400 μL of the S-trap binding buffer with centrifugation at 4000× *g* for 30 s in between each wash. Protein digestion was then performed via the addition of 125 μL of a digestion buffer, (50 mM TEAB containing a 1:25 w/w ratio of trypsin to protein) and incubated overnight at 37 °C. Peptides were eluted with 80 µL digestion buffer followed by 80 μL of 0.2% v/v aqueous FA and then 80 µL of 50% v/v aqueous ACN containing 0.2% v/v FA with centrifugation at 4000× *g* for 30 s. Eluents were combined, dried and then reconstituted in the loading buffer (2% v/v aqueous acetonitrile and 0.05% v/v trifluoroacetic acid) prior to LC-MS analysis.

### 3.8. Data-Dependent Acquisition (DDA) Proteomic Analysis

Peptides were analysed using an UltiMate 3000 nano (Thermo Fisher Scientific, San Jose, CA, USA) UHPLC system coupled with a timsTOF Pro mass spectrometer (Bruker Daltonics, Bremen, Germany) equipped with a CaptiveSpray ion source. Peptides were separated on an Aurora series UHPLC column (1.6 μm C18, 75 μm × 25 cm, IonOpticks, Fitzroy, VIC, Australia) with a flow rate of 0.4 μL/min, using a 120 min gradient of mobile Phase A (dH_2_O, 0.1% v/v FA) and mobile Phase B (ACN, 0.1% v/v FA). The gradient used was 2.0–17% B from 0 to 60 min, 17–25% B from 60 to 90 min, 25–37% B from 90 to 100 min and 37–95% B from 100 to 110 min, then held at 95% B for 1 min prior to ramping down to 2% B for 1 min and re-equilibration for 8 min. The capillary voltage was 1000 V, with a drying gas flow rate of 3.0 L/min and a drying temperature of 180 °C. A TIMS mobility range of 0.6–1.60 Vs/cm^2^ was used with an accumulation time of 100 ms. Peptides were detected in positive ionisation mode with parallel accumulation-serial fragmentation (PASEF)-DDA acquisition. Peptides were detected over a mass range of 100 to 1700 *m*/*z*.

### 3.9. Protein Identification and Label-Free Quantification

Raw file processing and label-free quantification (LFQ) was performed using MaxQuant 1.6.17.0. The protein sequence database for *E. coli* K-12 was obtained from UniProt (UP000000625, last modified 7 March 2021). This database and the reverse decoy were searched using MaxQuant. A fixed modification was carbamidomethyl-[C], and variable modifications included acetylation-[N] and oxidation [M]. The protein and peptide false discovery rate (FDR) had a threshold of 0.01. Other parameters were set at their default values. Matrix reduction was performed using Perseus 1.6.15.0. Proteins were filtered for those containing at least two peptides, one of which had to be unique. Zero filling was performed for proteins which were not detected in 3 out of 5 replicates from one group, using a value imputed by using one-third of the lowest LFQ value. Confidently identified proteins were mapped to biological pathways using the KEGG database and GOterms.

A schematic overview of the integrated ‘multi-omics’ workflow used in this study for lipidome and proteome analysis is shown in Supplementary Figure S1.

### 3.10. Statistical Analysis

Differences in the abundance of lipid species, and changes in protein abundances, were determined by an unpaired Student’s *t*-test correcting for multiple comparisons using the Holm–Šidák method. Volcano plots were generated and statistical significance was determined using GraphPad Prism 9.1.2 using a *p*-value ≤ 0.01 for lipids and 0.05 for proteins.

## 4. Conclusions

Adaptations to bacterial lipid metabolism play a key role in enabling increased resilience and survival upon exposure to environmental stress, as well as in the development of antibiotic or antimicrobial peptide resistance. The results reported here, which involve decreased total lipid abundance, an increased mol% abundance of cardiolipin and global remodelling of lipid acyl chain structures in response to *E. coli* nutrient depletion stress, highlight the need to use advanced lipidome analysis strategies such as FAIMS and UVPD-MS/MS for a detailed characterisation of alterations in the lipid metabolism and the structures that occur under such conditions. Furthermore, the analysis of temporal changes in the abundance of associated lipid metabolism enzymes, determined using complementary multi-omics proteomic analysis techniques, provided additional information and mechanistic insights into the metabolic changes responsible for, or that contribute to, environmental stress resistance responses and bacterial survival mechanisms.

## Data Availability

The data presented in this study are available on request from the corresponding author.

## Supplementary Materials

The following supporting information can be downloaded at: https://www.mdpi.com/article/10.3390/metabo12020171/s1, Table S1: Full names and abbreviations of the enzymes shown in Figure 5 and Figure 6, Table S2: Internal lipid standard composition and concentrations, Figure S1: Schematic overview of the multi-omics workflow used for lipidome and proteome analysis, Figure S2: Negative ionization mode nESI-FAIMS-MS analysis (*m*/*z* 600–800) of *E. coli* grown under EGP conditions, Figure S3: Negative ionization mode CID-MS/MS of the [M−H]^−^ precursor ion of PE(38:2) (*m*/*z* 770.56) from E. coli grown under EGP conditions, Figure S4: Negative ionization mode CID-MS/MS of the [M−H]^−^ precursor ion of PE(38:2) (*m*/*z* 770.56) from *E. coli* grown under SGP conditions, Figure S5: Negative ionization mode 213 nm UPVD-MS/MS for structural characterization of the [M−H]^−^ precursor ion of E. coli PG(37:2) observed at *m*/*z* 787.5514 in Figure 2 under SGP conditions, as predominantly containing PE18:1_19:1(c11Δ), Figure S6: Venn diagram of the number of proteins identified via DDA-nUHPLC-MS/MS proteomic analysis of E. coli grown under EGP versus SGP conditions.
