# Supplementary material for: Multi-Omic Analysis to Characterize Metabolic Adaptation of the E. coli Lipidome in Response to Environmental Stress
Source: Metabolites. 2022 Feb 11;12(2):171. doi: 10.3390/metabo12020171 (PMC8880424; doi:10.3390/metabo12020171)
Supplement: Supplementary file 1 [file metabolites-12-00171-s001.zip › metabolites-1579373-supplementary.pdf]

Supplemental Material

Multi-omic Analysis to Characterize Metabolic Adaptation of the *E. coli* Lipidome in Response to Environmental Stress

Thomas Kralj[1Δ], Madison Nuske[1 Δ], Vinzenz Hofferek[1], Marc-Antoine Sani[1], Tzong-Hsien Lee[2] Frances Separovic[1], Marie-Isabel Aguilar[2], Gavin E Reid[1,3*]

[1]School of Chemistry, Bio21 Molecular Science and Biotechnology Institute, The University of Melbourne, Melbourne, Victoria, 3010, Australia

[2]Department of Biochemistry and Molecular Biology, Monash University, Clayton VIC 3800, Australia

[3]Department of Biochemistry and Pharmacology, The University of Melbourne, Melbourne, Victoria, 3010, Australia

Keywords: *E.coli*; Lipidome; Proteome; Environmental Stress; Mass Spectrometry

Δ These authors contributed equally

* Corresponding Author:

e-mail: gavin.reid@unimelb.edu.au

Supplemental Tables

Supplemental Table S1. Full names and abbreviations of the enzymes shown in Figures 5 and 6.

Name	Abbreviation
Aerobic glycerol-3-phosphate dehydrogenase	GlpD
Cardiolipin synthase B	ClsB
Bifunctional protein Aas [Includes: 2-acylglycerophosphoethanolamine acyltransferase]	Aas
1-acyl-sn-glycerol-3-phosphate acyltransferase	PlsC
Anaerobic glycerol-3-phosphate dehydrogenase subunit A	GlpA
Anaerobic glycerol-3-phosphate dehydrogenase subunit B	GlpB
Anaerobic glycerol-3-phosphate dehydrogenase subunit C	GlpC
Cardiolipin synthase A	ClsA
Cardiolipin synthase C	ClsC
CDP-diacylglycerol pyrophosphatase	Cdh
CDP-diacylglycerol--glycerol-3-phosphate 3-phosphatidyltransferase	PgsA
CDP-diacylglycerol--serine O-phosphatidyltransferase	PssA
Diacylglycerol kinase	DgkA
Ethanolamine ammonia-lyase heavy chain	EutB
Ethanolamine ammonia-lyase light chain	EutC
Ethanolamine utilization protein EutA	EutA
Glycerol-3-phosphate acyltransferase	PlsB
Glycerol-3-phosphate dehydrogenase [NAD	GpsA
Glycerophosphodiester phosphodiesterase, cytoplasmic	UgpQ
Glycerophosphodiester phosphodiesterase, periplasmic	GlpQ
Lysophospholipase L2	PldB
Phosphate acyltransferase	PlsX
Phosphatidate cytidylyltransferase	CdsA
Phosphatidylglycerophosphatase A	PgpA
Phosphatidylglycerophosphatase B	PgpB
Phosphatidylglycerophosphatase C	PgpC
Phosphatidylserine decarboxylase proenzyme	Psd
Phospholipase A1	PldA
Probable glycerol-3-phosphate acyltransferase	PlsY
Thioesterase 1/protease 1/lysophospholipase L1	TesA
Uncharacterized protein YnbB	YnbB
Cyclopropyl Fatty Acid Synthase	Cfa
Acetyl-coenzyme A carboxylase carboxyl transferase subunit alpha	AccA
Biotin carboxyl carrier protein of acetyl-CoA carboxylase	AccB

3-oxoacyl-[acyl-carrier-protein] synthase 2	FabF
Acetyl-coenzyme A carboxylase carboxyl transferase subunit beta	AccD
3-hydroxyacyl-[acyl-carrier-protein] dehydratase FabZ	FabZ
3-hydroxydecanoyl-[acyl-carrier-protein] dehydratase	FabA
3-oxoacyl-[acyl-carrier-protein] synthase 3	FabH
3-oxoacyl-[acyl-carrier-protein] synthase 1	FabB
3-oxoacyl-[acyl-carrier-protein] reductase FabG	FabG
Biotin carboxylase	AccC
Enoyl-[acyl-carrier-protein] reductase [NADH] FabI	FabI
Malonyl CoA-acyl carrier protein transacylase	FabD
Acetyl-coenzyme A synthetase	Acs
Acyl-coenzyme A dehydrogenase	FadE
Acyl-CoA thioesterase 2	TesB
3-ketoacyl-CoA thiolase FadA	FadA
2,3-dehydroadipyl-CoA hydratase	PaaF
3-phenylpropionate/cinnamic acid dioxygenase ferredoxin--NAD	HcaD
3-ketoacyl-CoA thiolase FadI	FadI
Aldehyde-alcohol dehydrogenase [Includes: Alcohol dehydrogenase	AdhE
Acetyl-CoA acetyltransferase	AtoB
Alcohol dehydrogenase, propanol-preferring	AdhP
Fatty acid oxidation complex subunit alpha [Includes: Enoyl-CoA hydratase/3-hydroxybutyryl-CoA epimerase]	FadJ
Fatty acid oxidation complex subunit alpha [Includes: Enoyl-CoA hydratase/Delta]	FadB
Long-chain-fatty-acid--CoA ligase	FadD
Probable acetyl-CoA acetyltransferase	YqeF
Probable alcohol dehydrogenase	YiaY
S-(hydroxymethyl)glutathione dehydrogenase	FrmA
Medium-chain fatty-acid--CoA ligase	FadK
Long-chain acyl-CoA thioesterase	FadM
Alpha-galactosidase	MelA
Aldehyde reductase YahK	YahK
Glycerate 2-kinase	GarK
Glycerate 3-kinase	GlxK
Glycerol dehydrogenase	GldA
Glycerol kinase	GlpK
PEP-dependent dihydroxyacetone kinase, ADP-binding subunit DhaL	DhaL
PEP-dependent dihydroxyacetone kinase, dihydroxyacetone-binding subunit DhaK	DhaK
PEP-dependent dihydroxyacetone kinase, phosphoryl donor subunit DhaM	DhaM
Beta-galactosidase	LacZ

Acyl-CoA thioester hydrolase	YciA
7alpha-hydroxysteroid dehydrogenase	HdhA
Thioredoxin/glutathione peroxidase	BtuE
Disulfide-bond oxidoreductase YghU	YghU
Catalase HPII	KatE
Peroxiredoxin OsmC	OsmC
Uncharacterized protein YnjA	YnjA
Thiol peroxidase	Tpx
Alkyl hydroperoxide reductase C	AhpC
Probable cytochrome c peroxidase	Ccp
Catalase-peroxidase	KatG
Superoxide dismutase [Mn]	SodA
Deferrochelatase/peroxidase EfeB	EfeB
Dye-decolorizing peroxidase YfeX	YfeX
Adenine deaminase	AdeD
Superoxide dismutase [Fe]	SodB
Superoxide dismutase [Cu-Zn]	SodC
Hydroxylamine reductase	Hcp
Thioredoxin reductase	TrxB
Glutathione S-transferase YfcF	YfcF
Disulfide-bond oxidoreductase YfcG	YfcG
Peroxiredoxin Bcp	Bcp
Glutathione reductase	Gor

Supplemental Table S2. Internal lipid standard composition and concentrations.

Lipid Standards	Cat No.	Lipid Class	Concentration (μM)
PC(15:0/18:1(d7))	791637C	PC	66.4
PE(15:0/18:1(d7))	791638C	PE	843.9
PS(15:0/18:1(d7))	791639C	PS	77.2
PG(15:0/18:1(d7))	791640C	PG	209.4
PI(15:0/18:1(d7))	791641C	PI	23.6
PA(15:0/18:1(d7))	791642C	PA	87
LPC(18:1(d7))	791643C	Lyso PC	18.9
LPE(18:1(d7))	791644C	Lyso PE	10.3
LPS(13:0)	858140P	Lyso PS	20.9
LPG(13:0)	858126C	Lyso PG	107.7
PC(p18:0/18:1(d9))	852475C	PC	6.4
PE(p18:0/18:1(d9))	852474C	PE	67.6
MG(18:1(d7))	791646C	MG	27.5
DG(15:0/18:1(d7))	791647C	DAG	17
TG(15:0/18:1(d7)/15:0)	791648C	TAG	24.6
SM(d18:1/18:1(d9))	791649C	SM	27.1
Cer(d18:1(d7)/15:0)	860681P	Cer	18.8
CE(18:1(d7))	791645C	ChlE	15.2
Cholesterol(d7)	700041P	Chl	50.8
CL(14:0/14:0/14:0/14:0)	710332C	CL	39.2

PC: glycerophosphatidylcholine; PE: glycerophosphatidylethanolamine; PS: glycerophosphatidylserine; PG: glycerophosphatidylglycerol; PI: glycerophosphatidylinositole; PA: glycerophosphatidic acid; LPC: lyso-glycerophosphatidylserine; LPE: lyso-glycerophosphatidylethanolamine; LPS: lyso-glycerophosphatidylserine; LPG: lyso-glycerophosphatidylglycerol; PC(p): plasmalogen PC; PE(p): plasmalogen PE; MG: monoacylglycerol; DG: diacylglycerol; TG: triacylglycerol; SM: sphingomyelin; Cer: Ceraminde; CE: cholesterol ester; CL: cardiolipin.

Supplemental Figures

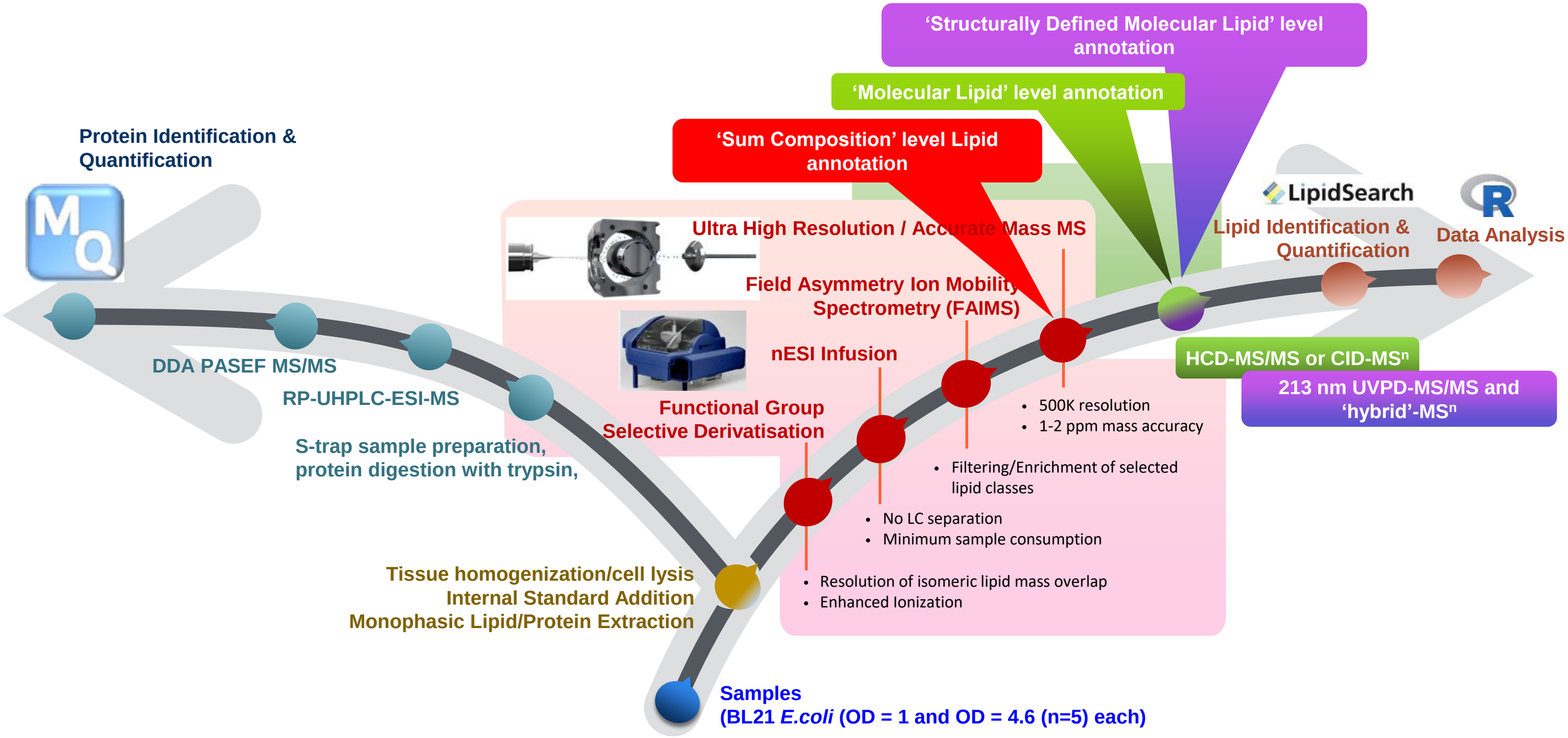

Figure S1. Schematic overview of the multi-omics workflow used for lipidome and proteome analysis.

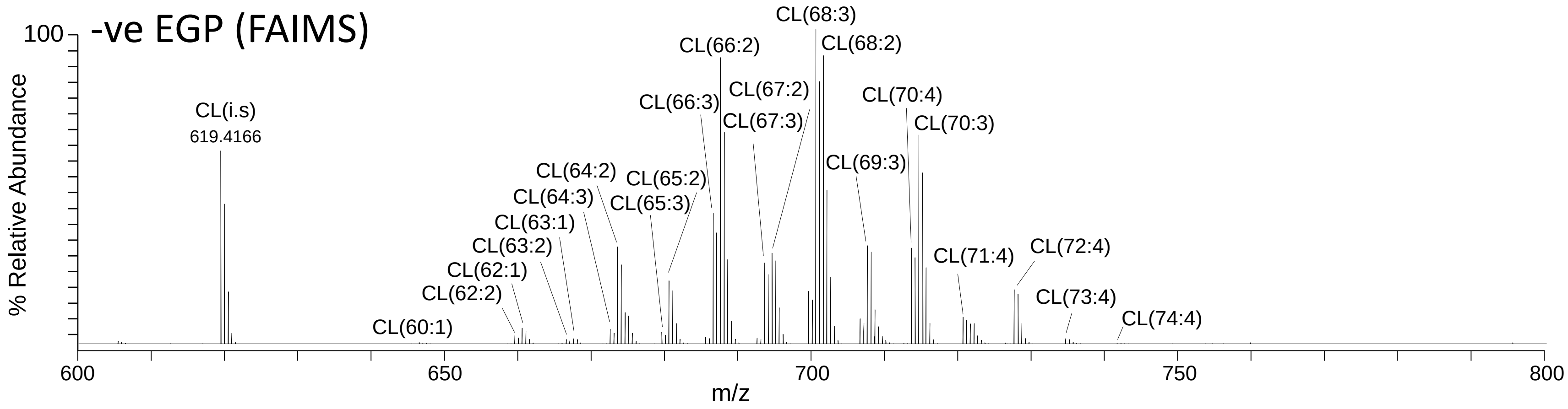

Figure S2. Negative ionization mode nESI-FAIMS-MS analysis (m/z 600-800) of *E. coli* grown under EGP conditions.

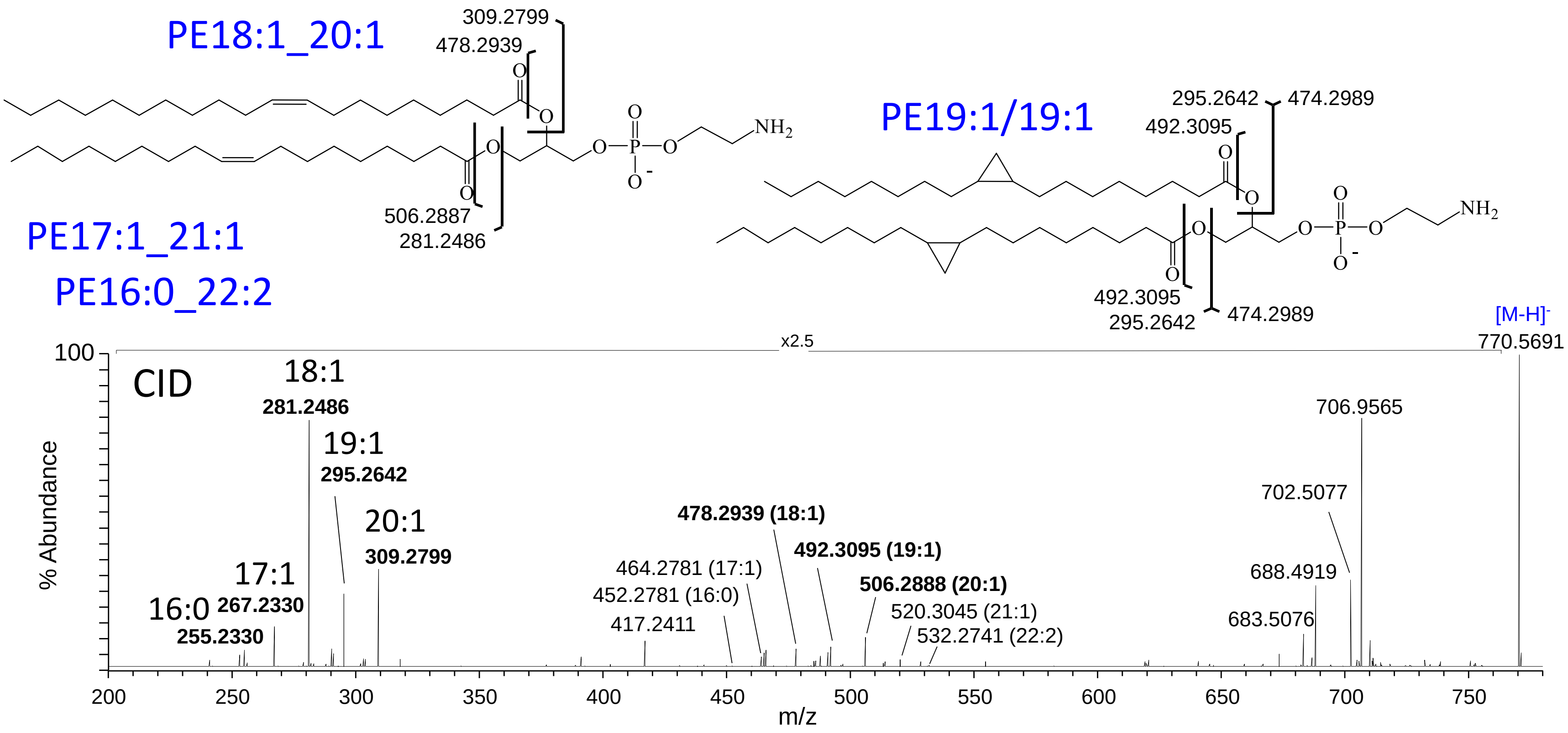

Figure S3. Negative ionization mode CID-MS/MS of the [M-H]$^-$ precursor ion of PE(38:2) (m/z 770.56) from *E. coli* grown under EGP conditions.

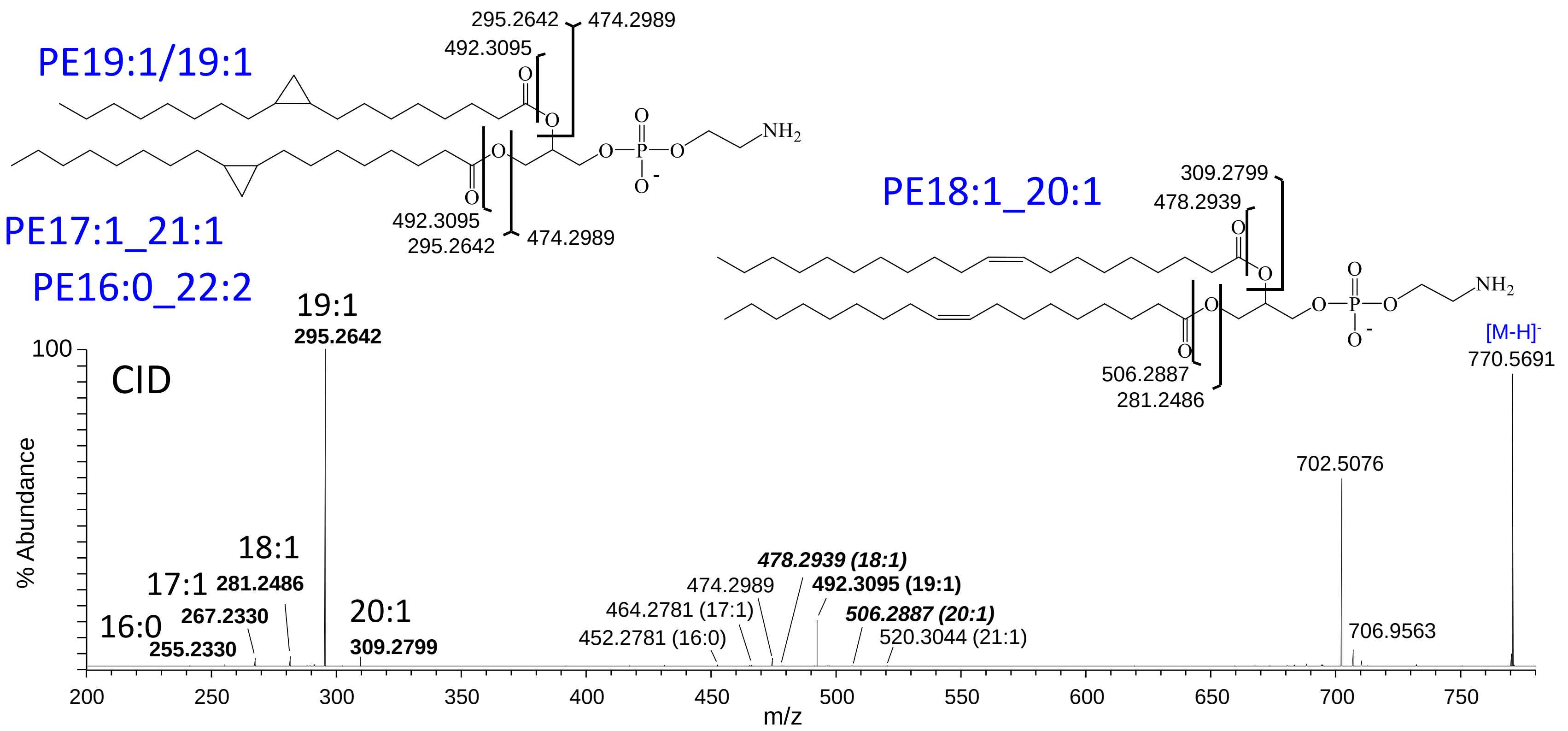

Figure S4. Negative ionization mode CID-MS/MS of the [M-H]$^-$ precursor ion of PE(38:2) (m/z 770.56) from *E. coli* grown under SGP conditions.

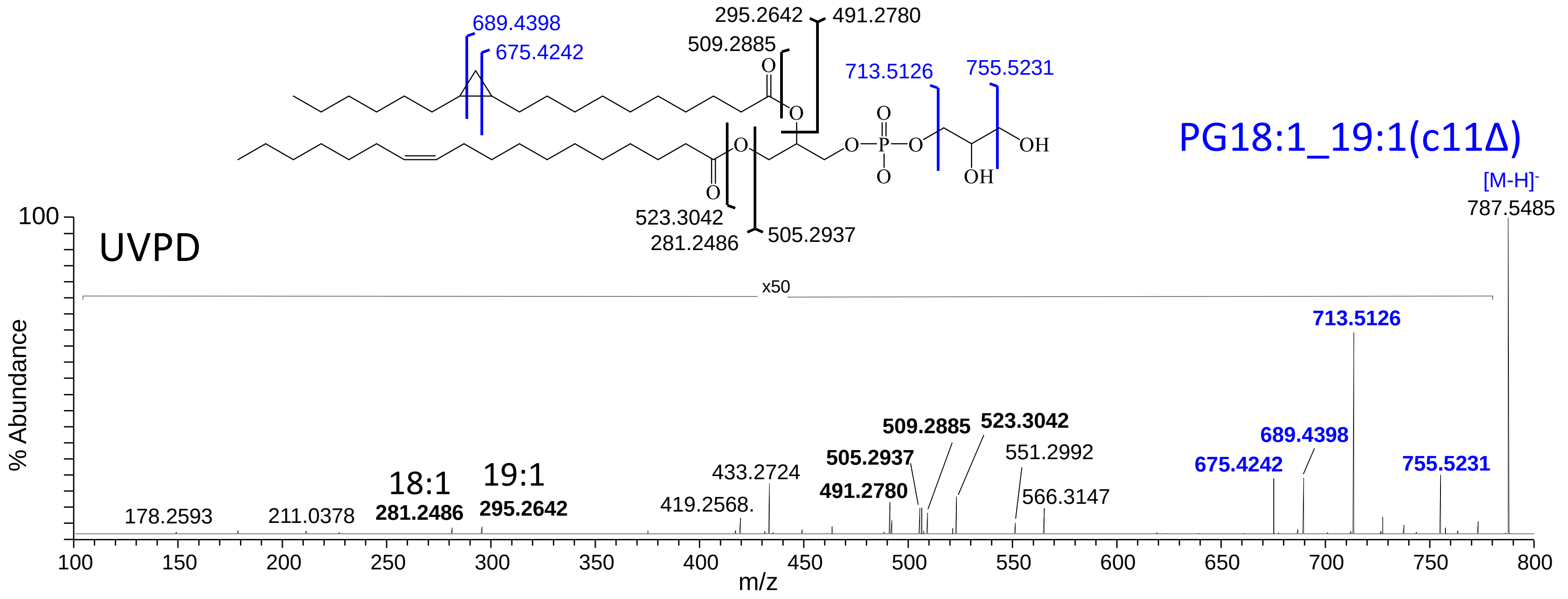

Figure S5. Negative ionization mode 213 nm UPVD-MS/MS for structural characterization of the [M-H]$^-$ precursor ion of *E. coli* PG(37:2) observed at m/z 787.5514 in Figure 2 under SGP conditions, as predominantly containing PE18:1_19:1(c11Δ). The structure shown in the inset indicates the assigned bond cleavage sites for the major product ions. Product ions labelled in blue text are unique to UVPD.

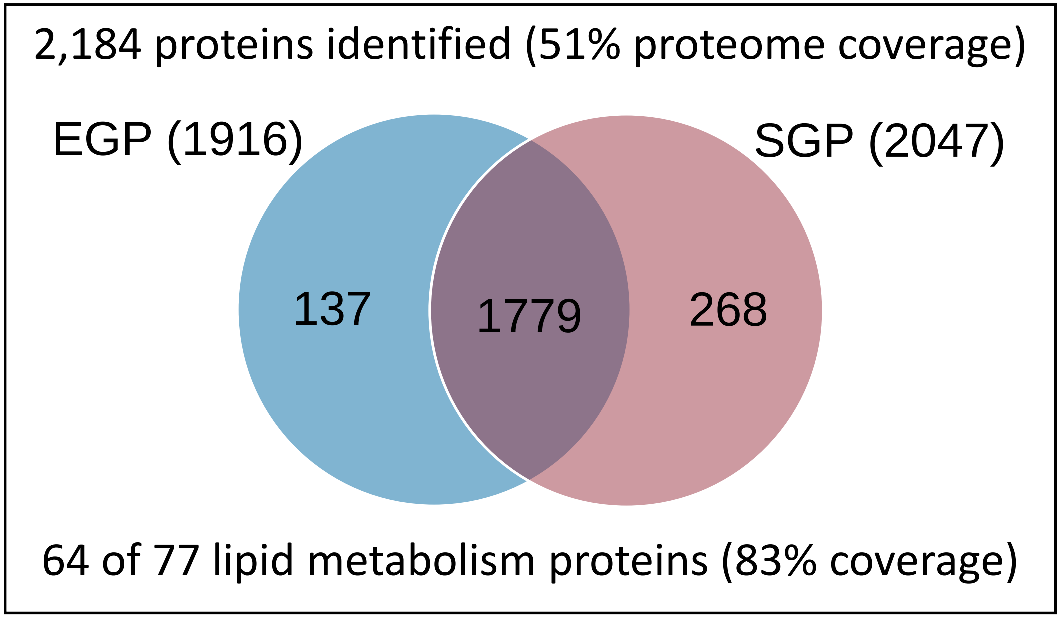

Figure S6. Venn diagram of the number of proteins identified via DDA-nUHPLC-MS/MS proteomic analysis of *E. coli* grown under EGP versus SGP conditions.
